# Near-Field Chipless Radio-Frequency Identification (RFID) Sensing and Identification System with Switching Reading

**DOI:** 10.3390/s18041148

**Published:** 2018-04-09

**Authors:** Ferran Paredes, Cristian Herrojo, Javier Mata-Contreras, Miquel Moras, Alba Núñez, Eloi Ramon, Ferran Martín

**Affiliations:** 1 GEMMA/CIMITEC, Departament d’Enginyeria Electrònica, Universitat Autònoma de Barcelona, 08193 Bellaterra, Spain; cristian.herrojo@uab.cat (C.H.); FranciscoJavier.Mata@uab.cat (J.M.-C.); ferran.martin@uab.es (F.M.); 2 Institut de Microelectrònica de Barcelona, IMB-CNM (CSIC), 08193 Bellaterra, Spain; Miquel.Moras@imb-cnm.csic.es (M.M.); alba.nunez@imb-cnm.csic.es (A.N.); Eloi.Ramon@imb-cnm.csic.es (E.R.)

**Keywords:** chipless-RFID, split-ring resonators (SRRs), microwave sensors

## Abstract

A chipless radio-frequency identification (chipless-RFID) and sensing system, where tags are read by proximity (near-field) through a switch, is presented. The tags consist of a set of identical resonant elements (split-ring resonators or SRRs), printed or etched at predefined and equidistant positions, forming a linear chain, each SRR providing a bit of information. The logic state (‘1’ or ‘0’) associated with each resonator depends on whether it is present or not in the predefined position. The reader is an array of power splitters used to feed a set of SRR-loaded transmission lines (in equal number to the number of resonant elements, or bits, of the tag). The feeding (interrogation) signal is a harmonic (single-tone) signal tuned to a frequency in the vicinity of the fundamental resonance of the SRRs. The set of SRR-loaded lines must be designed so that the corresponding SRRs are in perfect alignment with the SRRs of the tag, provided the tag is positioned on top of the reader. Thus, in a reading operation, as long as the tag is very close to the reader, the SRRs of the tag modify (decrease) the transmission coefficient of the corresponding reader line (through electromagnetic coupling between both SRRs), and the amplitude of the output signal is severely reduced. Therefore, the identification (ID) code of the tag is contained in the amplitudes of the output signals of the SRR-loaded lines, which can be inferred sequentially by means of a switching system. Unlike previous chipless-RFID systems based on near-field and sequential bit reading, the tags in the proposed system can be merely positioned on top of the reader, conveniently aligned, without the need to mechanically place them across the reader. Since tag reading is only possible if the tag is very close to the reader, this system can be also used as a proximity sensor with applications such as target identification. The proposed chipless-RFID and sensing approach is validated by reading a designed 4-bit tag. For identification purposes, this system is of special interest in applications where a low number of bits suffice, and tag reading by proximity is acceptable (or even convenient). Applications mostly related to secure paper, particularly involving a limited number of items (e.g., exams, ballots, etc.), in order to provide authenticity and avoid counterfeiting, are envisaged. As a proximity sensor, the system may be of use in detecting and distinguishing different targets in applications such as smart packaging.

## 1. Introduction

Radio-frequency identification (RFID) is a wireless technology useful in many applications, including identification, tracking, sensing, and security, among others [1,2]. Typically, RFID tags are equipped with an antenna (for communication with the reader) and a silicon integrated circuit (IC), or chip, where information is stored. Although chipped-RFID tags are relatively inexpensive (typically several Euro cents), in many applications this cost may represent a significant percentage of the item price, and this prevents this technology from fully expanding in many sectors.

To alleviate the cost-related limitation of chipped-RFID, much effort has been dedicated to the so-called chipless-RFID in the last decade, chips being replaced with printed encoders [3,4,5,6,7,8]. Such encoders, typically implemented on plastic substrates by means of printing techniques, such as inkjet printing (or massive manufacturing processes, such as rotogravure, screen printing, etc.), using conductive inks, are low-cost when compared to silicon ICs. However, printed encoders occupy a significant area (proportional to the number of bits), and their data storage capability is small when compared to the 96 bits of regulated passive RFID technology at Ultra-High Frequency (UHF) band. Additionally, the read ranges in chipless-RFID are small when compared to those achievable in RFID systems with tags equipped with chips (several meters in passive UHF-RFID and dozens of meters in systems that use active tags). Therefore, many efforts have been focused in recent years on improving the data storage capacity and read distances of chipless-RFID systems.

There are two main approaches to the implementation of chipless-RFID systems: those based on the time domain [9,10,11,12,13,14,15,16,17,18], and those based on the frequency domain [3,4,19,20,21,22,23,24,25,26,27,28,29,30,31,32,33,34,35,36,37,38,39]. Most systems within the former approach are based on time-domain reflectometry (TDR), where the identification (ID) code is given by the echoes generated by the tag (a delay line with reflectors at certain positions) to a narrow pulse (interrogation signal). Despite the fact that TDR tags implemented on surface acoustic wave (SAW) technology are competitive [9,12,14,15,16], such tags are not compatible with standard printing processes. On the other hand, attempts to implement TDR-based tags on fully planar technology have been successful, but the achieved number of bits has been very limited [11,13,17].

Frequency-domain-based chipless-RFID systems use printed tags consisting of a set of resonant elements, each tuned to a different frequency. In such tags, each resonator typically provides a bit of information, ‘1’ or ‘0’ depending on whether the resonator is functional or detuned, and the interrogation signal is a multi-frequency signal that must cover the whole spectral bandwidth occupied by the tag. The ID code is given by the presence or absence of singularities at the predefined frequencies of the different tag resonators, contained either in the transmission coefficient (retransmission-based tags [19,20]) or in the radar cross section (backscattered tags [21,27]). In these tags, the number of bits is limited by the achievable density of bits per frequency, which is related to the spectral bandwidth of each resonant element. The required frequency sweep of the interrogation signal is proportional to the number of bits. Consequently, it is not possible to exceed certain limits with a simple and low-cost reader.

To partially alleviate the limited data storage capability of spectral signature barcodes (as such frequency-domain-based tags are usually designated), hybrid encoding, i.e., exploiting several domains simultaneously, has been proposed. Examples include frequency/phase deviation [32], frequency/notch bandwidth [39], frequency/notch magnitude [38], and frequency/peak magnitude [21], among others. By this means, more than one bit of information per resonant element has been demonstrated, but the reported tags are far from the data storage capability of chipped tags.

In References [40,41,42,43,44,45], a new approach for the implementation of chipless-RFID systems, based on near-field and sequential bit reading, was proposed. This is a time-domain approach, but, rather than in the echoes of a pulsed signal, the ID information is contained in the envelope of an amplitude modulated signal generated by the tag when this is displaced over the reader, in close proximity to it. The tag is a set of identical resonators forming a linear chain. The resonant elements are etched at predefined and equidistant positions in the tag substrate, and the logic state ‘1’ or ‘0’ is determined by the functionality of the resonant element (similar to spectral signature barcodes). The reader is a transmission line fed by a harmonic (single tone) carrier signal tuned to a frequency in the vicinity of the resonance frequency of the tag resonators. Thus, by displacing the resonant elements of the tag sequentially over the transmission line, in close proximity to it, line-to-resonator coupling with those functional resonators of the tag arises, thereby modulating the transmission coefficient at the carrier frequency. Therefore, the amplitude of the carrier (feeding) signal is modulated, and the ID code is contained in the envelope function, which can be inferred from an envelope detector. The functionality of this near-field chipless-RFID system has been validated by reading tags with 40 bits [44], and the robustness has been demonstrated by reading tags implemented on plastic substrates through conductive inks [41], and also on paper substrates [45].

The previous chipless-RFID system requires the tag to be mechanically guided over the reader (for sequential tag reading). In this paper, we propose a new time-domain chipless-RFID sensing and identification system, conceptually similar to the previous one, where tag reading does not require tag motion. In this new system, the tags are identical to those of the near-field chipless-RFID, i.e., linear chains of identical resonators (present or not, at predefined and equidistant positions), but tag reading merely requires the proper positioning of the tag over the active part of the reader (a guide system may be used for that purpose). Since tag reading requires proximity, the proposed system may be useful as a proximity sensor with identification capability. As compared to optical barcodes, the main advantage of the proposed system is the fact that, although tags can be photocopied, such copied tags cannot be read with the proposed reader. Therefore, the system provides protection against counterfeiting.

## 2. The Proposed Chipless-RFID Sensing and Identification System

**Figure 1:**
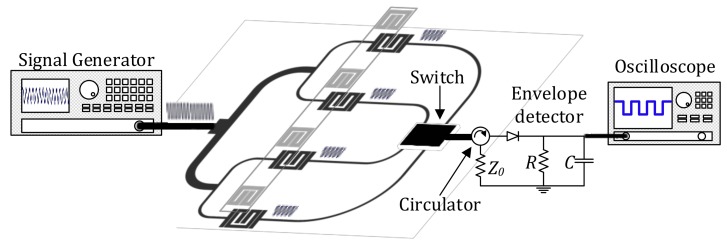
Sketch of the proposed chipless radio-frequency identification (RFID) sensing and identification system. The envelope detector is preceded by a circulator (configured as an isolator) in order to avoid mismatching reflections from the diode (a highly nonlinear device). To ensure proper alignment between the reader and the tag, a guiding system may be used.

A sketch of the proposed chipless-RFID sensing and identification system is depicted in Figure 1. The interrogation signal, with frequency *f_c_*, is injected into the input port of a microstrip line, which divides the signal into as many channels as the number of bits in the considered tags (four in our case). In each channel, the sensing (reading) element is a transmission line loaded with a split-ring resonator (SRR) in band-pass configuration (i.e., the SRRs are coupled with the input and output access lines through gap capacitors, providing a band-pass functionality). The tag is a linear chain of SRRs identical to those of the reader, but oppositely oriented in order to favor the coupling between tag and reader SRRs. With an identical shape, the coupling between both resonators (those of the reader and those of the tags) is maximized. By positioning the tag on top of the reader (with the SRRs of the tag face-to-face to those of the reader in close proximity), the electromagnetic coupling between the SRRs of the lines and the functional SRRs of the tag modifies the transmission coefficient of the corresponding lines, effectively modifying (decreasing) the amplitude of the feeding signal at the output ports of such lines. Note that this modulation is only effective in those channel lines with a functional SRR on top of it. Therefore, the ID code is contained in the amplitudes of the output signals of the different channels, with high- and low-level amplitudes corresponding to the ‘0’ and ‘1’ logic states, respectively. It is important to highlight that the frequency of the feeding signal, *f_c_*, must be selected in the band pass of the frequency response of the unloaded channel lines (i.e., without SRR tags on top of them). The reason for this is that, with this selection, we obtain a significant decrease in the transmission coefficient at *f_c_* when the SRR of the tag is situated on top of the SRR of the line. Consequently, a high amplitude contrast between the ‘0’ and ‘1’ logic states is obtained, as required in order to have a reasonable tolerance margin against possible misalignments between the tag and the reader and to increase the vertical detection distance (typically up to 1 mm). It is worth mentioning that the tag can be put in contact with the reader if the SRRs are etched in the opposite side of the substrate.

For tag reading, a switch is considered, as shown in Figure 1, in order to read the tag sequentially. The output port of the switch is connected to an envelope detector, which provides the amplitude of the output port of each channel line and consequently the ID code of the tag. Such codes can only be detected (read) if the tag is within the influence of the electromagnetic fields generated by the channel lines (i.e., a distance in the order of 1 mm). This means that the proposed system can be used as a proximity detector with identification functionality, that is, we can detect the presence of a target in a certain position (very close to the reader or detector) and identify it.

**Figure 2:**
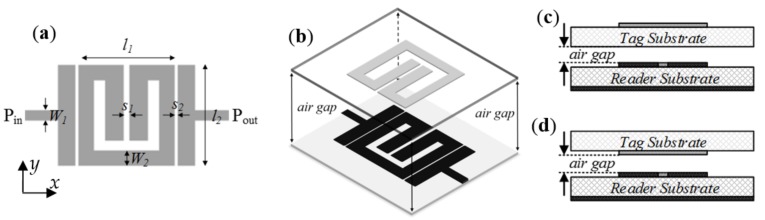
(**a**) Layout of the channel lines, consisting of a split-ring resonator (SRR)-loaded microstrip line with SRR coupled to the input and output ports through capacitive gaps (relevant dimensions are indicated); (**b**) perspective view of the channel line with tag on top of it; (**c**) cross section view of the channel line with tag on top of it and SRR tags etched on the top side of the substrate (further away from the reader); (**d**) section view of the channel line with tag on top of it and SRR tags etched on the bottom substrate side (closer to the reader).

**Figure 3:**
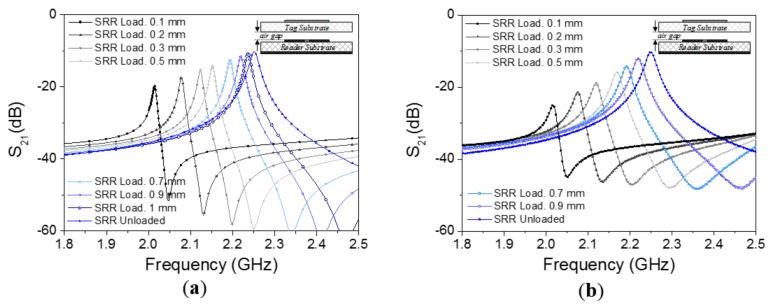
Frequency responses of the channel line without tag on top of it and with SRR tags (perfectly aligned) at different distances. The considered configuration is the one of Figure 2c; (**a**) electromagnetic simulation; (**b**) measurement. The dimensions are: *W*_1_ = 0.58 mm, *W*_2_ = 0.87 mm, *l*_1_ = 5.53 mm, *l*_2_ = 5.86 mm, *s*_1_ = 0.35 mm, *s*_2_ = 0.2 mm. The considered tag substrate has a dielectric constant of *ε_r_* = 10.2 and a thickness of *h* = 0.63 mm.

**Figure 4:**
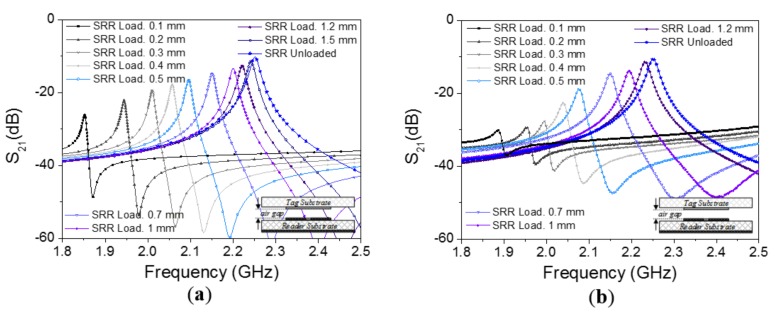
Frequency responses of the channel line without tag on top of it and with SRR (perfectly aligned) at different distances. The considered configuration is that of Figure 2d; (**a**) electromagnetic simulation; (**b**) measurement. The dimensions and tag substrate are those indicated in the caption of Figure 3.

The layout of the channel lines (all identical) is similar to the one considered in [44,45] for the implementation of a near-field chipless-RFID system with sequential bit reading capabilities through tag motion (see Figure 2a). Such a layout has been optimized in order to obtain a selective bandpass response with a central frequency of 2.25 GHz (see Figure 3). The considered substrate is the *Rogers RO3010* with dielectric constant of *ε_r_* = 10.2 and thickness of *h* = 0.635 mm. By situating an oppositely oriented SRR on top of the line SRR, face-to-face to it, as the perspective view of Figure 2b indicates, the electromagnetic coupling between both SRRs modifies the transmission coefficient. The simulated responses for different distances between the line and SRR tags (air gap) are also depicted in Figure 3, where it can be seen that the transmission coefficient at *f_c_* experiences a significant displacement up to roughly 1 mm separation. In the results of Figure 3, the SRR of the tag is etched in the upper (top) side of the tag substrate (Figure 2c). By etching the SRRs on the other (bottom) side of the tag substrate (Figure 2d), the system is also functional. In this case, the read distance increases (Figure 4), but it is not possible to contact the tag with the reader; if the tag and lines are put in direct connection a short-circuit occurs (an undesired situation).

**Figure 5:**
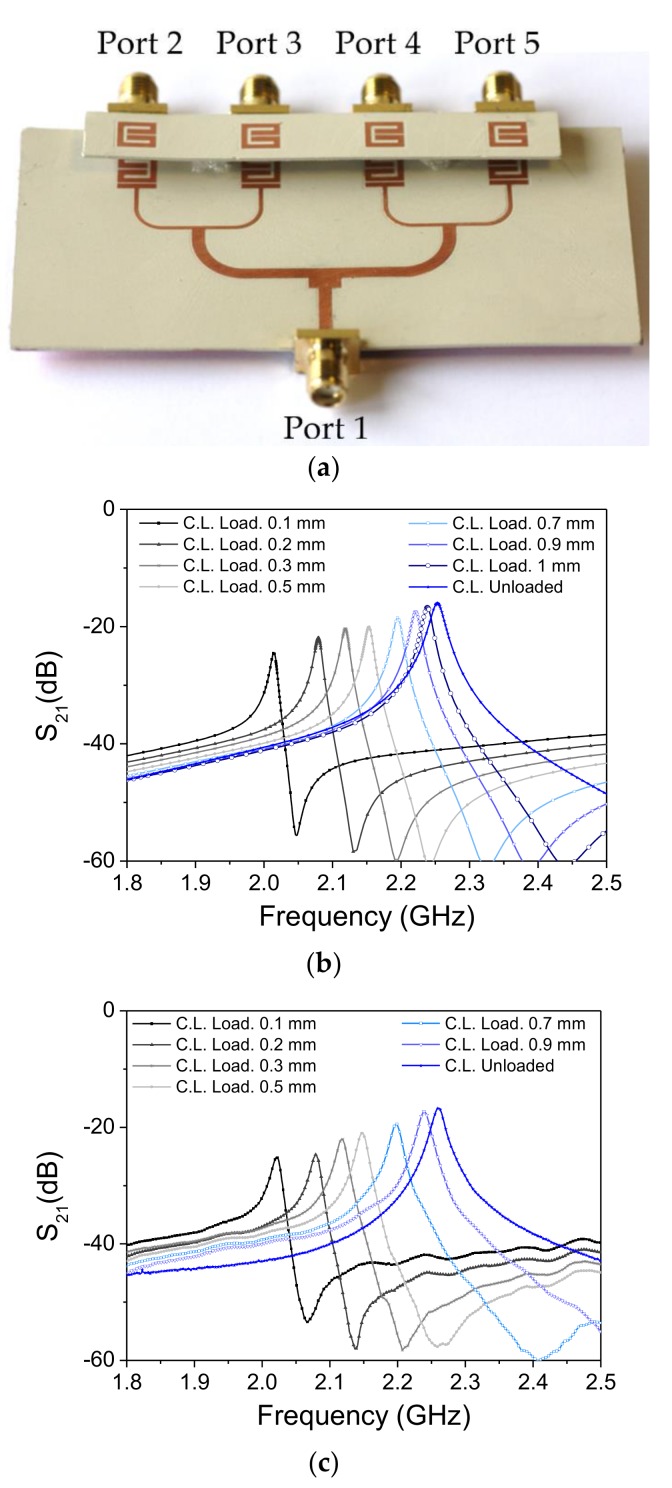
Frequency responses of the channel line without tag on top of it and with SRR tags (perfectly aligned) at different distances. The considered configuration is that of Figure 2c; (**a**) photograph of the prototype with tag on top and face up; (**b**) electromagnetic simulation; (**c**) measurement.

**Figure 6:**
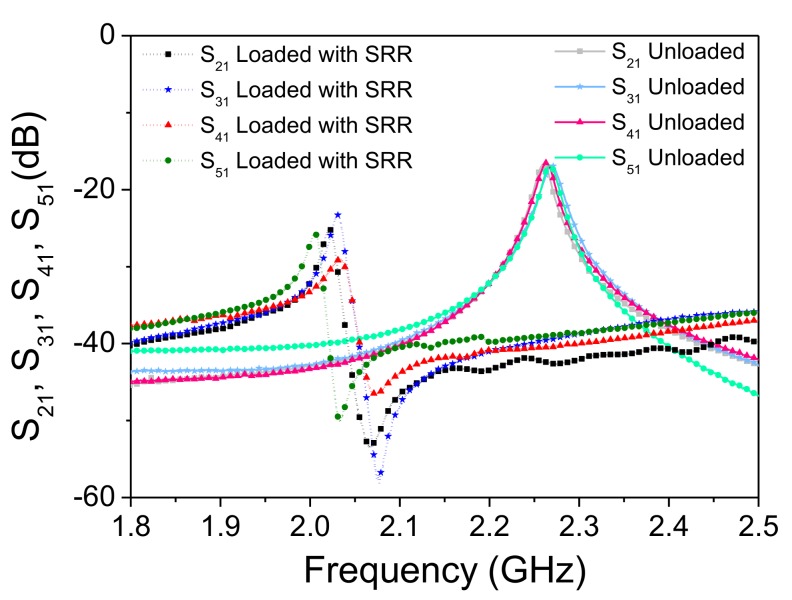
Transmission coefficient for the different channel lines with and without SRR tags on top of them.

Let us now analyze the response of one of the SRR-loaded lines in the whole system, with a tag with all resonators functional on top of it (and face up). The structure is depicted in Figure 5a, whereas the simulated responses, for different air gap distances, are shown in Figure 5b. Note that, in this case, the maximum transmission for each response is roughly 6 dB lower (as compared to Figure 3), corresponding to an output power level divided by four. This is an expected value on account of the power splitting of the T-junctions. Nevertheless, it is demonstrated that by tuning the feeding signal in the vicinity of the frequency of maximum transmission for the unloaded reader (2.25 GHz), the displacement experienced by the transmission coefficient is significant up to air gaps close to 1 mm, thanks to the presence of the transmission zero in the responses. Note that the optimum situation in terms of dynamic range is the one corresponding to an air gap of roughly 0.5 mm, since in this case, the transmission zero is close to 2.25 GHz, and the displacement experienced by the transmission coefficient with and without the SRR tag on top of the reader line is larger than 40 dB. The measured responses, depicted in Figure 5c, are in agreement with the simulated ones. It is worth mentioning that the responses of Figure 5 correspond to the transmission coefficient between the input and the output of port 2. Nevertheless, similar responses are obtained for the transmission coefficients of the other output ports. To demonstrate this, Figure 6 depicts the responses of the different transmission coefficients for the reader without the tag, and with an all-functional SRR tag, situated at a distance (air gap) of 0.1 mm. As can be seen, the two sets of responses are very similar, indicating that the behavior of each channel is roughly the same.

**Figure 7:**
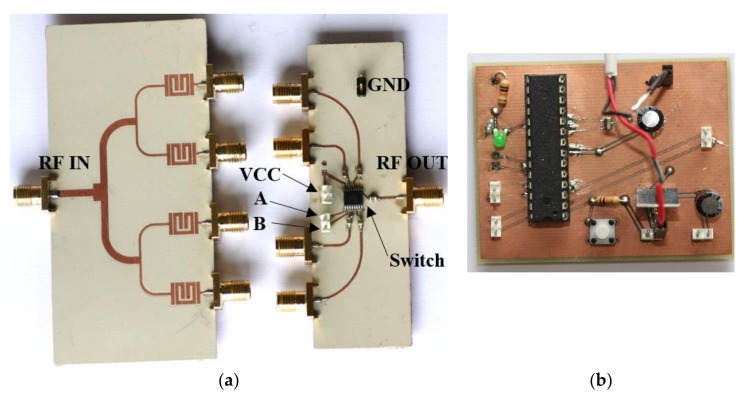
(**a**) Photograph of the power divider and switch; (**b**) photograph of the control circuit. The switch is managed by the microcontroller, which is responsible for carrying out a sequential scan of the channel lines as well as for powering the switch.

For tag reading, a switching scheme is considered, where each reader channel is sequentially selected, and the corresponding signal is driven to the output port of the switch, which is in turn connected to the input port of the envelope detector. The switch is based on the *Analog Devices HMC241AQS16E* integrated circuit. A photograph of the switch circuit is shown in Figure 7a. The switch requires two supply pins (VCC and GND), as well as pins A and B to select the switch input channels. In order to select the switch output ports automatically, as well as to manage the switching time, an *Atmel ATmega328P* microcontroller and the necessary electronic components, disposed in a printed circuit board (PCB) were used (see Figure 7b). Note that the bits are read sequentially, in a known order. Because the orientation of the SRRs of the tag and reader must be opposite, it ensures that the tags are correctly read out.

## 3. System Validation

**Figure 8:**
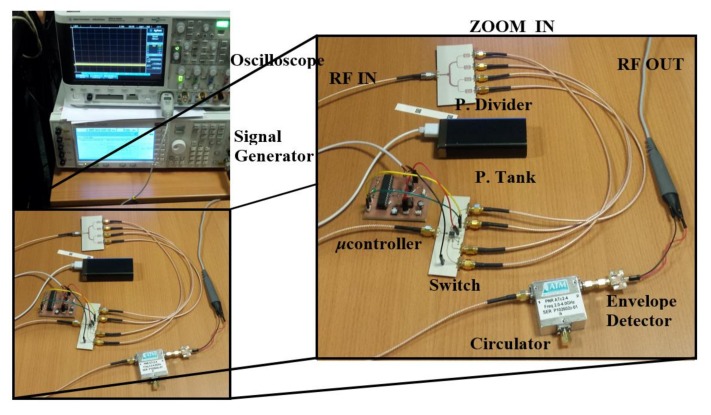
Photograph of the chipless setup, where all components are shown.

**Figure 9:**
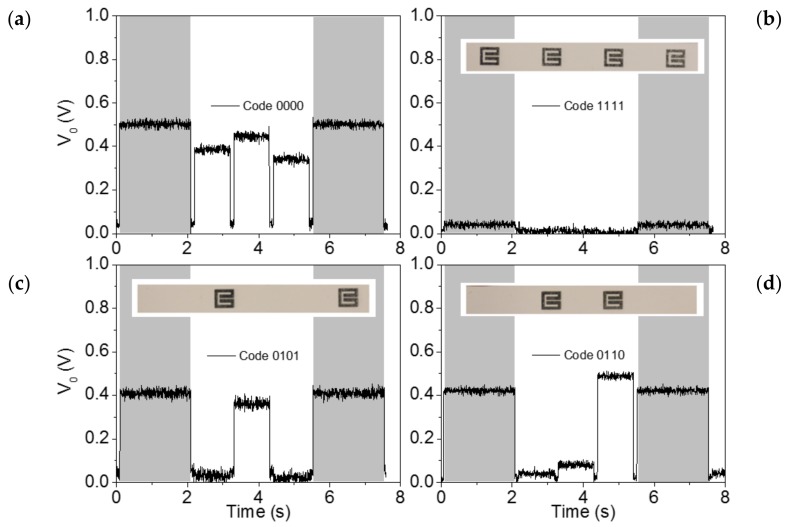
Envelope functions of the tags with the indicated codes. The considered codes are: (**a**) ‘0000’; (**b**) ‘1111’; (**c**) ‘0101’; (**d**) ‘0110’. These responses were obtained by leaving the tag to rest on top of the reader lines. The gray vertical stripes show the first channel line, where the switch time has been configured to 2 s.

The whole chipless setup, used for experimental validation, is shown in Figure 8. The *Agilent E44338C* signal generator is used to feed the power divider with a harmonic signal (whose amplitude is reduced by 6 dB at each channel line of the power divider, for the reasons explained before). The switch, managed by a microcontroller and powered by an Universal Serial Bus (USB) power tank, sequentially redirects the RF input channel lines to an RF output. To be able to discern the channel line position, the microcontroller is programmed to wait 0.1 s between channels sweep, and the switch time is configured to take 2 s at the first channel line and 1 s for the rest of the lines (see Figure 9, where the gray vertical stripes indicate the 2 s time of the first channel). By this means, the initiation of the bit sequence is clearly identified. The switch RF output is connected to the envelope detector, which is indeed preceded by an isolator (implemented by means of the *L3 Narda-ATM ATc4-8* circulator), in order to prevent unwanted reflections from the diode, a highly nonlinear device. The diode used to obtain the envelope function is the *Avago HSMS-2860*, whereas the necessary low-pass filter is implemented by means of the *Agilent N2795A* active probe (with resistance and capacitance *R* = 1 MΩ and *C* = 1 pF, respectively), connected to an oscilloscope (model *Agilent MSO-X-3104A*).

In order to validate the system, a chipless tag was fabricated using one layer of *Dupont^TM^ PE410* Ag conductive ink (thickness of 2.6 µm and conductivity of 7.28 × 10^6^ S/m) inkjet printed on *PowerCoat^TM^ HD ultra-smooth* paper with a dielectric constant of *ε_r_* = 3.1 and a thickness of *h* = 230 µm (the *Ceradrop Ceraprinter X-Serie* inkjet printer was used). We used paper as substrate and conductive ink for tag printing in order to imitate a real scenario, where these kinds of systems might be applied to direct code printing onto the items (e.g., smart packaging). Switching reading was first applied in the absence of a tag, which is equivalent to a tag without resonators (with code ‘0000’). From this response (see Figure 9a), it is reasonable to set the threshold level to differentiate the two logic states to 0.2 V. Further tag readings, corresponding to different codes with SRRs face up, are included in Figure 9, and it can be seen that the obtained responses validate the proposed approach. 

**Figure 10:**
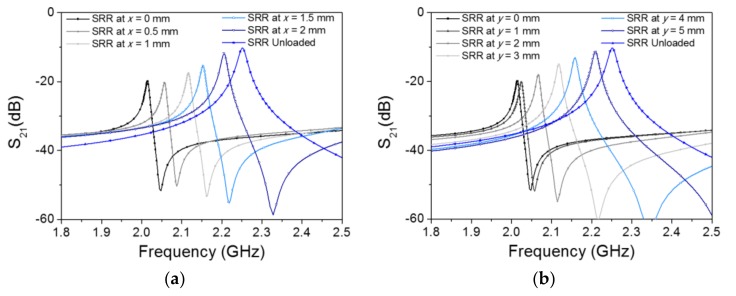
Electromagnetic simulation of the transmission coefficient for the channel line with SRR on top of it and the indicated longitudinal (**a**) and lateral (**b**) misalignments.

One aspect that may limit the correct tag reading is the longitudinal and lateral misalignment between the SRR of the tag and that of the reader. This aspect is difficult to evaluate experimentally because of the difficulty of accurately measuring such misalignments, but it can be estimated with an electromagnetic simulation. Thus, Figure 10 depicts the simulated responses of one SRR over one channel line for different longitudinal and lateral displacements (the air gap set to 0.1 mm). As can be seen, a significant displacement in the transmission coefficient is maintained up to 2 mm misalignment in the *x*-direction (longitudinal) and up to 5 mm misalignment in the *y*-direction (lateral). Therefore, these results point towards the fact that the proposed system is quite robust when dealing with lateral and longitudinal misalignments between the tag and the reader.

We would like to mention that, as compared to the near-field chipless-RFID systems reported in References [40,41,42,43,44,45], the main advantage of the system proposed in this paper is the fact that once the tag is positioned over the reader, a mechanical displacement for tag reading is not necessary. Nevertheless, scaling up this system to many bits is difficult, and therefore the system should be focused on applications requiring a limited number of bits. However, by introducing additional splitters into the reader, tags with a higher number of bits (e.g., 8-bit tags) can be read. Alternatively, it is possible to replace the splitters of the reader with a switch (or a set of switches) in order to increase the number of bits, thus avoiding excessive complexity on the reader side. In this case, the number of switch outputs must be equal to the number of inputs of the switch prior to the circulator, and equal to the number of bits. However, this is a higher cost solution if at least two switches are involved.

## 4. Conclusions

In conclusion, a novel chipless-RFID system, where the tags are read by proximity through near-field coupling, and the bits are sequentially inferred from a switch, has been proposed in this paper. The validity of the system has been demonstrated by considering 4-bit tags consisting of chains of SRRs inkjet printed on a paper substrate. Previous chipless-RFID systems based on near-field coupling and sequential bit reading in the time-domain require the tags to be mechanically placed over the reader for tag reading; in the system reported in this paper, tag motion is not needed. Time division multiplexing is carried out by means of a switch, managed by a microcontroller, which sequentially provides the logic state of each bit. Such a logic state is in turn provided by the amplitude of the signal present at the output port of each channel (or bit) line of the reader (inferred form an envelope detector). Because tag reading proceeds by proximity, the system is also useful as a proximity sensor with identification capability.
